# Lingering health-related anxiety about radiation among Fukushima residents as correlated with media information following the accident at Fukushima Daiichi Nuclear Power Plant

**DOI:** 10.1371/journal.pone.0217285

**Published:** 2019-05-31

**Authors:** Chihiro Nakayama, Osamu Sato, Minoru Sugita, Takeo Nakayama, Yujiro Kuroda, Masatsugu Orui, Hajime Iwasa, Seiji Yasumura, Rima E. Rudd

**Affiliations:** 1 Department of Public Health, Fukushima Medical University School of Medicine, Fukushima, Japan; 2 Children's Department of Welfare Faculty, Fukushima College, Fukushima, Japan; 3 Department of Environmental and Occupational Health, Toho University School of Medicine, Tokyo, Japan; 4 Department of Health Informatics, Kyoto University School of Public Health, Kyoto, Japan; 5 Harvard T.H. Chan School of Public Health, Department of Social and Behavioral Sciences, Boston, Massachusetts, United States of America; University of New South Wales, AUSTRALIA

## Abstract

Following the March 2011 accident at Fukushima Daiichi Nuclear Power Plant, many residents of Fukushima have faced anxieties about the health impacts of radiation exposure. Considering that source of information may influence resident anxiety, this study aimed to elucidate the correlation between the two. In addition, a health literacy query was included to examine a possible relationship between anxiety and health literacy skills. A mail survey was conducted in August 2016 among 2000 residents of Fukushima Prefecture aged 20 to 79 years. Survey items included questions about current health anxieties caused by radiation, trusted sources of information about radiation, and media used to obtain information on radiation. The survey valid response rate was 43.4%. Results of multiple linear regression analysis revealed that anxiety was significantly higher for the groups indicating “trust in citizen groups” and “use of internet sites.” Anxiety was significantly lower for the groups indicating “trust in government ministries,” “trust in local government,” and “use of local broadcast television.” Also anxiety was significantly lower for groups with higher health literacy. It was found that the significant relationship to anxiety varies depending on the sources of trust and media used. There is a possibility that this was caused by the difference between the contents of each information and media reports. In preparation for any future nuclear accident, government may consider action to improve the media literacy of residents. In addition, improving health literacy of both the recipient and the sender of information can improve access to information and thereby safeguard the health and well-being of the public.

## Introduction

Following the Great East Japan Earthquake of March 11, 2011, the accident at Fukushima Daiichi Nuclear Power Plant caused massive quantities of radioactive materials to be released and spread across a wide area. Since then, many Fukushima residents have developed anxiety about the effects of radiation exposure on their health [1]. With the exception of the area surrounding the nuclear power plant, current air radiation doses in most areas of Fukushima have fallen to levels similar to those of major world cities, at roughly 0.1μSv/h [2]. Moreover, external exposure in the first four months after the accident was below 1 mSv for 94% of those Fukushima Prefecture residents who experienced it [3]. Internal exposure for 95% of those who experienced the accident was also below the detection limit [4], which 1/10 to 1/100 lower than exposure levels caused by the Chernobyl nuclear accident [5, 6]. The inspection of all rice produced in Fukushima since 2015 found zero cases exceeding the standard allowable radiation (100 becquerels/kg) [7], and the quantity of radioactive material contained in meals consumed by general Fukushima households has also been confirmed to be extremely low, at less than 1 becquerel/kg [8]. Still, Fukushima residents are exhibiting deeply rooted anxiety about their health due to radiation exposure [9, 10].

The emotional effects on residents following the Three Mile Island and Chernobyl nuclear accidents are well documented [11, 12, 13, 14]. Excessive anxiety has been hypothesized to lower immunocompetence [15]. Impairment of resident mental and physical health is also a concern. Consequently, inquiries into the state of resident anxiety and its causes could offer insight for rebuilding resilience and for the design of preventive efforts.

### Background

A body of literature indicates that media plays an important role not only in providing information but also in shaping perceptions. Content as well as volume of coverage are important considerations. For example, Kasperson et al. (1988) [16] developed a theoretical framework to demonstrate that information about contemporary disasters obtained from the media has a major effect on risk perception, i.e., a human being’s subjective estimation of risk. Furthermore, research has indicated that anxiety can actually be reduced by an intervening perception that the risk is dangerous but manageable [17]. Renn et al. (1992) demonstrated that an expanded volume of media reporting tends to heighten risk perception [18]. Vestermann et al. (1999) showed that massive press coverage following a major catastrophe increases anxiety among individuals [19].

Several studies have examined the correlation between risk perception, anxiety, information, and the mass media following the Fukushima nuclear accident. However, none of these studies has focused specifically on the residents of Fukushima Prefecture including evacuation zone. This study is the first to focus on Fukushima Prefecture residents to determine how anxiety about radiation has been associated with information source and the media.

Sugimoto et al. (2013) surveyed 1560 residents of Soma City in July 2011 and found that radiation/health fears were high among those who used word-of-mouth, or rumors, as a means to obtain information. In addition, survey findings indicated that fear for the future was low among users of national newspapers and high among users of local newspapers. Furthermore, this research effort found that fear about social disruption was high among radio listeners [20]. In a survey of the headlines of national newspapers published between the date of the earthquake (March 11, 2011) and January 2012, Kanda et al. (2014) identified many reports about “danger and risk” in March 2011. Kanda and colleagues noted that although the circumstances of radiation exposure remained unclear; these reports were important for disseminating information necessary for risk avoidance. This study also suggests the possibility that these reports may have had a subsequent impact on increasing risk perception among the general population [21].

The Fukushima nuclear accident was the first nuclear disaster in the world to occur since the widespread proliferation of the internet. Needless to say, the internet became an important source of information for residents of Fukushima [22, 23]. A December 2015 online survey of 9249 residents of Tokyo, Osaka, and Fukushima, Murakami et al. (2016) found that those who trusted the government had a low “dread” risk perception according to Slovic’s classification, while those who trusted TV and radio, direct information from friends, and online information from researchers and others had a high “dread” risk perception [24]. In contrast, Rubin et al. (2012) examined 284 citizens of the UK who were in Japan at the time of the nuclear accident and found that anxiety levels were high among people who obtained information from Japanese government websites and blogs [25]. Another study conducted in Belgium, only marginally related, found that risk perception about the Fukushima accident was higher among television viewers and consumers of word of mouth, but lower among those who were satisfied with media coverage of Fukushima and who had been exposed to the issue for an extended period of time [26].

Against this backdrop, the present study aimed to examine a possible link between health-related anxiety and residents’ trusted sources of information. Considered in this study were additional factors such as demographics, knowledge, and health literacy skills.

## Methods

This study is based on a survey of residents of Fukushima Prefecture. The study methods were approved by the Fukushima Medical University’s Ethics Committee (Approval number: 2699). We considered a returned questionnaire as participant consent to the objective of the study and their voluntary participation in it. This is the second study focused on data from a postal based survey questionnaire of a sample of residents of Fukushima Prefecture. Kuroda et.al published a paper based on the same data set in 2018[27]. The first study focused on differences among residents in evacuated and non-evacuated areas and, in particular on any association between health literacy and anxiety related to radiation. This current study focuses on sources of information and any association between sources of information and reported anxiety related to radiation.

### Participants

This survey targeted 2000 residents of Fukushima Prefecture aged 20 to 79. We divided Fukushima Prefecture into four areas based on the general regional classification of Aizu, Nakadōri, Hamadōri, and the evacuation area (the restricted area, evacuation prepared area, and deliberate evacuation area as determined on April 22, 2011), and selected 500 people from each area. The selection was based on a two-stage stratified random sampling (stage one survey of region, stage two of individuals selected randomly from the Basic Resident Registration). Nakadōri and Hamadōri included local municipalities that were partially in the evacuation area; these were included in the evacuation area. The instrument used in the present study, entitled “Survey of Health and Information,” was administered as an anonymous, self-reporting postal questionnaire. We considered a returned questionnaire as participant consent to the objective of the study and their voluntary participation in it. We received 916 responses from 1985 survey subjects (excluding those returned to sender because no one was residing at the address). After excluding 55 respondents who left age or sex blank, we analyzed data from 861 respondents, for a valid response rate of 43.4%.

### Survey instrument

For demographic information, respondents were asked to report their age, sex, area of residence, as well as current residence status. Options included: own home, public housing, government subsidized housing, rental home or apartment, temporary housing, home of friend/relative or other. Respondents were asked to specify whether they had children aged 18 or younger, 19 or above, were pregnant, had a pregnant family member, or had “none” (no children) at the time of the earthquake. Survey participants were also asked about their educational background, employment status, family structure, housing prior to the earthquake, whether or not they had relocated to avoid radiation.

Questions also included indications of social capital and social participation as well as health related behaviors such as exercise, sleep satisfaction, alcohol consumption, and smoking. Respondents were asked to rate their health status on a five-point scale (“extremely good,” “very good, “good,” “fair,” and “not healthy”). Participants completed the health literacy scale developed by Ishikawa et al. for use with the general public [28]. The Cronbach’s alpha coefficient of the scale has been reported as 0.86, and in the present study sample it was 0.89. Knowledge about radiation was assessed according to a respondent’s knowledge of the following five areas: properties of radiation, probability of death from cancer, genetic impact, DNA repair, and food reference values. Participants were asked to rate short sentences which were well known and often misunderstood as true or false, and correct answers scored one point each for a total of five questions. The Cronbach’s alpha for knowledge about radiation was 0.52.

A specific group of health related questions focused on anxiety. Participants were asked to rate their level of health anxiety immediately after the nuclear accident and current level about the effects of radiation on their health due to the nuclear accident on a five-point scale ranging from “None” to “Extreme”. Questions asked about receiving health examinations or attending lectures (8 items). Participants were asked to rate their radiation anxiety (the 7-item Radiation Anxiety Scale developed by Fukasawa et al. [29]). The Cronbach’s alpha coefficient of the scale has been reported as 0.81, and in the present study sample it was 0.84. Participants were asked about actions to protect against radiation (4 items). They were asked to comment on the impact of harmful rumor and indicate something gained through the earthquake experience such as posttraumatic growth which can be seen as positive psychological change experienced as a result of the struggle with a major life crisis or a traumatic event.

Several questions focused on sources of information to trust. Survey participants were asked to select up to three main items from the following 11 options: International organizations (UN, WHO, etc.), experts from universities and other academic institutions, government ministries, local newspapers, national newspapers, NHK (public broadcasting), private local broadcast television, private national broadcast television, local government, volunteer organization such as citizen groups, and “none of the above.” Participants were asked to indicate media used for information about radiation by selecting up to three main items from the following 13 options: Local newspapers, national newspapers, NHK television (public broadcasting), private local broadcast television, private national broadcast television, radio, internet news, internet sites/blogs, SNS (Facebook, Twitter, etc.), magazines/books, local government publications, word of mouth, and “none of the above.”

### Analysis plan

Many of the survey answers were collapsed in two or three categories for analysis. For example, **f**or age, respondents were divided into three groups comprising those aged 20 to 44 years (prime adults), 45–64 years (middle aged), and 65 years and older (elderly). For area, two groups were created, and comprised those in the evacuation area or ‘other’ (Aizu, Nakadōri and Hamadōri). Respondents specified whether they had children and, for analysis, they were divided into those with and without children.

Respondents were asked to rate their health status on a five-point scale (“extremely good,” “very good, “good,” “fair,” and “not healthy”) and were then divided into the two groups of ‘healthy’ (those who responded “extremely good,” “very good, and “good,”) and ‘not so healthy’ (those who responded “fair” or “not healthy”). For health literacy, scores are determined as the sum of the values of the five items, with those above the second tertile placed in the “high” group and those in the second tertile or below placed in the “low” group. Scores of knowledge about radiation were determined as the sum of the values of the five items. Those above the second tertile placed in the “high” group and those in the second tertile or below placed in the “low” group

Participants were asked to rate “Your current level of anxiety about the effects of radiation on your health due to the nuclear accident” on a five-point scale ranging from “None” to “Extreme,” with responses treated as a continuous variable ranging from 1 to 5. The objective variable was “Your current level of anxiety about the effects of radiation on your health due to the nuclear accident”. The explanatory variables were“Trusted sources of information about radiation,” and “Media used for information about radiation”. Respondents were asked to select the three main sources/media,

In order to consider the correlation between “current anxiety” and all other items, we first performed univariate analysis (Student’s t-test, and variance analysis when there were multiple categories for explanatory variables), with “current anxiety” as the objective variable and all other responses as explanatory variables. P<0.05 was considered statistically significant. Next, multiple regression analysis was performed using explanatory variables found to be significant in the univariate analysis, with “current anxiety” as the objective variable. Of the explanatory variables found to be significant, we excluded responses of “none of the above” for trusted information source. Ultimately, health status, health literacy score, residing at one’s own home, having no children, and working were used as moderator variables. Finally, age, sex, area, and knowledge score on radiation were forcefully input as basic moderator variables.

Questions about the trusted source of information and media used for information about radiation elicited multiple answers from participants (who were instructed to choose three main items). Due to the restriction that "Please select up to 3 main items", these explanation variables are not independent of each other completely. Therefore, we created six multiple regression models that included explanatory variables one by one. However, we did not see the confounding of "trusted information sources" and "media to use" to avoid complexity. Thus, variables found to be significant through univariate analysis, i.e., “trust in government ministries,” “trust in local government,” “trust in citizen groups,” “use of private local broadcast television,” “use of internet sites/blogs,” and “use of word of mouth,” were applied in different six models to examine their associations with current anxiety levels.

## Results

According to demographics, it is estimated that the average age of 20 to 79 years old in Fukushima prefecture as a whole is about 51.6 years old. The ratio of males and females is 49.5% for males and 50.5% for females. Study samples are largely representative of established demographics for age and gender. Mean age (years ± SD) of respondents was 56.4±14.8 years for all. Participant characteristics are summarized in Table 1. Mean level of current anxiety was 2.47 ± 1.09.

**Table 1:** Basic information on respondents (the collapsed data and current radiation anxiety).

Item	Category	n	%
Age	Prime adults (20–44 years)	202	23.5
	Middle aged (45–64 years)	336	39
	Elderly (65–80 years)	323	37.5
Sex	Male	382	44.4
Area	Evacuation area	192	22.3
	Other(Aizu, Nakadori, Hamadori)	669	77.7
Current residence	Residing at home	645	75.2
	Not residing at home	213	24.8
Children at time of earthquake	No	393	45.6
	Yes	468	54.4
Education	Two year college, vocational school or above	287	33.8
	High school	564	66.2
Employment	Working	505	59.5
	Not working	343	40.5
Current family structure	Single person household	106	12.4
	Married couple only	235	27.4
	Other	517	60.2
Health status	Not healthy	463	54
	Healthy	394	46
Health literacy	High	259	31.6
	Low	560	68.4
Radiation knowledge	High	267	32.1
	Low	565	67.9
Current radiation anxiety	1 None	199	23.3
	2 Just a little	213	25
	3 Some	328	38.4
	4 A lot	69	8.1
	5 Extreme	44	5.2

**Fig 1:**
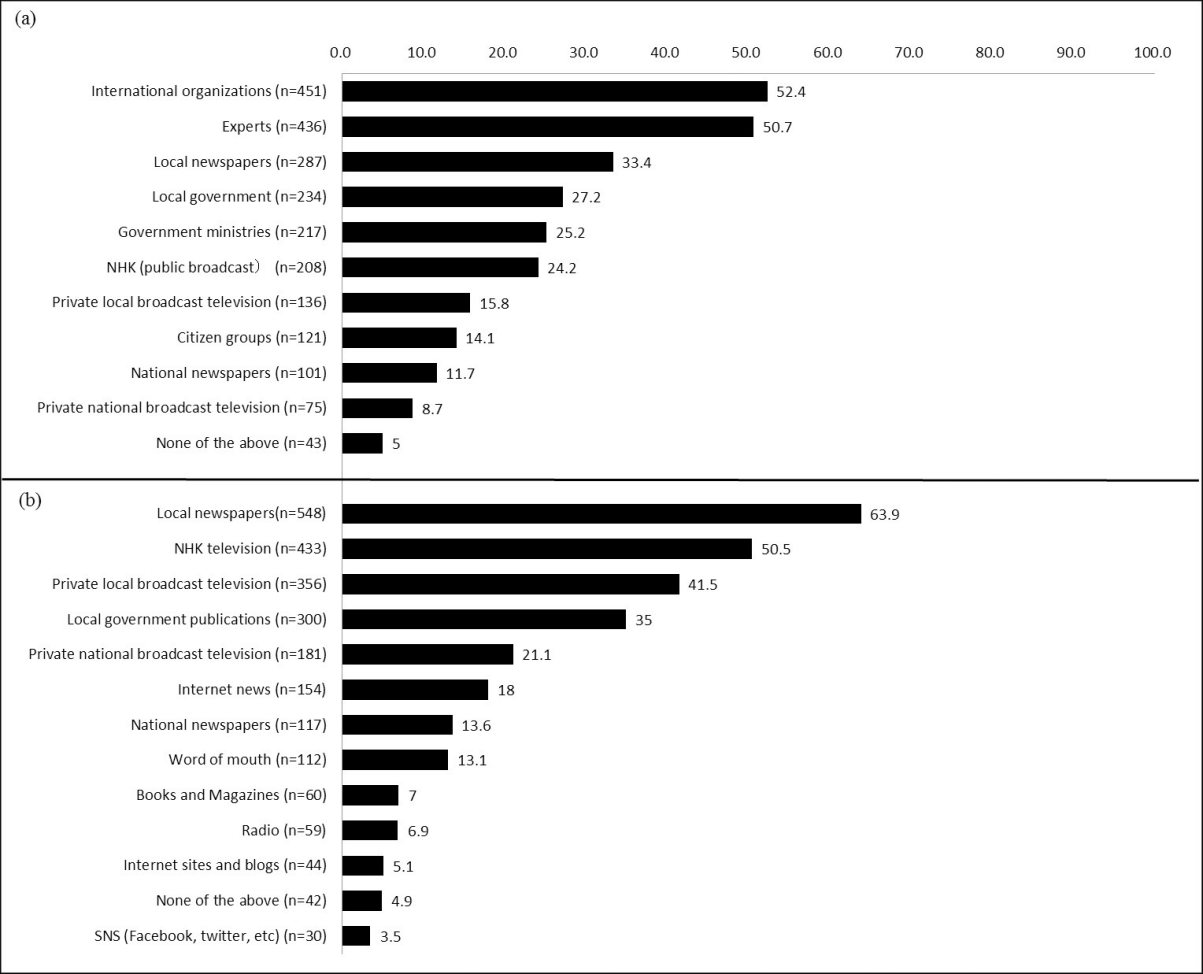
Proportion of (a) trusted information sources and (b) media used (% n = 861).

Participants’ trusted sources of information and media sources used by respondents are summarized in Fig 1. Participants were instructed to choose three main items, therefore the total is over 100 percent.

An analysis of trusted sources of information indicates that 52.4% trusted international organizations, 50.7% trusted experts, 27.2% trusted local governments, 25.2% trusted government ministries, and 14.1% trusted citizen groups. The examination of media sources used by respondents indicates that local newspapers were used by 63.9%, NHK television was used by 50.5%, private local broadcast television were used by 41.5%, word of mouth was used by 13.1%, and internet sites/blogs were used by 5.1%.

Results from the univariate analysis (t-test and ANOVA) are shown in Tables 2, 3, 4, and 5. Table 2 indicates Results of univariate analysis for demographic information on respondents with current anxiety as the response variable. Significantly higher anxiety was found among those respondents in the evacuation area compared to non-evacuation area (p = <0.001). Significantly lower levels of anxiety were also observed among those living in their own home, those without children and those who were working, relative to their counterparts who did not live in their own home, those with children, and those who were not working, respectively (p = 0.002, 0.022, 0.007). Significantly higher anxiety was also found among those who relocated to avoid radiation compared to those who did not relocate.

**Table 2:** Results of univariate analysis for demographic information on respondents with current anxiety as the response variable.

Item	Category	Mean score for current anxiety	P value
Area	Evacuation area	2.815	<0.001
	Non-evacuation area	2.369	
Current residence	Residing at home	2.396	0.002
	Not residing at home	2.664	
Children at time of earthquake	No	2.374	0.022
	Yes	2.546	
Education	Above High school	2.458	0.966
	High school or below	2.454	
Employment	Working	2.388	0.007
	Not-working	2.595	
Family structure	Single	2.356	0.283
	Married couple only	2.551	
	Other	2.456	
Pre-earthquake residence	Own home	2.474	0.618
	Not own home	2.423	
Relocation to avoid radiation	Yes	2.816	<0.001
	No	2.330	

**Table 3:** Results of univariate analysis for health and lifestyle on respondents with current anxiety as the response variable.

Item	Category	Mean score for current anxiety	P value
Health status	Not healthy	2.689	<0.001
	Healthy	2.224	
Mean total score for health literacy	High	2.288	0.001
	low	2.548	
Mean total score for radiation knowledge	High	2.323	0.009
	low	2.532	
Sleep satisfaction	Very or fairly dissatisfied	2.768	0.001
	Slightly dissatisfied, satisfied	2.418	
Exercise	Less than once a week	2.510	0.139
	Once a week or more	2.387	
Alcohol consumption	Yes	2.437	0.601
	No	2.480	
Smoking	Yes	2.477	0.932
	No	2.469	
Social capital (Total of 4 questions)	9 or above	2.434	0.056
	8 or below	2.690	
Participation in local groups	No	2.495	0.669
	Yes	2.459	

**Table 4:** Results of univariate analysis for items related to radiation and anxiety on respondents with current anxiety as the response variable.

Item	Category	Mean score for current anxiety	P value
Radiation anxiety immediately after nuclear accident	Higher than average	3.266	<0.001
	Low	2.022	
Regular health checks at municipality, workplace	Yes	2.443	0.455
	No	2.499	
Complete physical	Yes	2.387	0.336
	No	2.484	
Individual dosimeter measurement of external radiation	Yes	2.733	0.017
	No	2.438	
WBC internal radiation measurement	Yes	2.694	0.001
	No	2.402	
Fukushima Health Management Survey	Yes	2.664	0.02
	No	2.429	
Thyroid test field information session	Yes	2.714	0.548
	No	2.466	
Local physician lecture on radiation	Yes	2.314	0.395
	No	2.474	
Other lecture or information session	Yes	2.367	0.457
	No	2.475	
Mean total score for radiation anxiety (Total score of 7-item)	Higher than average	3.046	<0.001
	Lower than average	1.823	
Radiation dose measurement	Yes	3.117	<0.001
	No	2.393	
Avoiding high radiation areas	Yes	2.912	<0.001
	No	2.193	
Attentive to food radiation and production area	Yes	3.008	<0.001
	No	2.225	
Purchase water	Yes	2.948	<0.001
	No	2.259	
Affected by harmful rumor	Yes	2.711	<0.001
	No	2.085	
Gained something through disaster experience	Yes	2.555	0.004
	No	2.333	

**Table 5:** Results of univariate analysis for trusted information sources and media used on respondents with current anxiety as the response variable.

Item	Category	Mean score for current anxiety	P value
Trusted information source	International organization	2.467	0.986
	Not international organization	2.468	
	Experts	2.477	0.789
	No experts	2.457	
	Government ministries	2.265	0.002
	Not government ministries	2.535	
	Local newspapers	2.373	0.076
	Not local newspapers	2.514	
	National newspapers	2.470	0.978
	Not national newspapers	2.467	
	NHK	2.367	0.13
	Not NHK	2.499	
	Local broadcast TV	2.309	0.065
	Not local broadcast TV	2.497	
	National broadcast TV	2.365	0.399
	Not national broadcast TV	2.477	
	Local government	2.319	0.015
	Not local government	2.523	
	Citizen groups	2.733	0.004
	Not Citizen groups	2.423	
	None of the above	3.000	0.001
	Not none of the above	2.439	
Media for obtaining information	Local newspapers	2.444	0.455
	Not local newspapers	2.502	
	National newspapers	2.388	0.414
	Not national newspapers	2.477	
	NHK TV	2.403	0.096
	Not NHK TV	2.527	
	Local broadcast TV	2.332	0.003
	Not local broadcast TV	2.560	
	National broadcast TV	2.556	0.207
	Not national broadcast TV	2.440	
	Radio	2.483	0.896
	Not radio	2.463	
	Internet news	2.575	0.166
	Not internet news	2.440	
	Internet sites/blogs	2.864	0.012
	Not internet sites/blogs	2.443	
	SNS	2.667	0.301
	Not SNS	2.457	
	Books and magazines	2.650	0.171
	Not books and magazines	2.451	
	Local government publications	2.379	0.092
	Not Local government publications	2.511	
	Word of mouth	2.750	0.003
	Not word of mouth	2.421	
	None of the above	2.524	0.718
	Not none of the above	2.462	

Table 3 indicates Results of univariate analysis for health and lifestyle on respondents with current anxiety as the response variable. Those with high health literacy had significantly lower anxiety compared to those with low health literacy (p = 0.001). Those with high radiation knowledge also had significantly lower anxiety (p = 0.009). Significantly higher anxiety was found among those who indicated that they were not so healthy and among those who indicated a dissatisfaction with sleep (p <0.001, = 0.001).

Table 4 indicates results of univariate analysis for items related to radiation and anxiety on respondents with current anxiety as the response variable. Significantly higher anxiety levels were noted for a variety of participants. Participants who were with higher than average anxiety immediately after the accident indicated higher anxiety compared to those who were with lower anxiety (p < 0.001). Significantly higher anxiety was also found among those who underwent individual dosimeter measurement of external radiation (p = 0.017), WBC internal radiation measurement (p = 0.001), Fukushima Health Management Survey (p = 0.020). In addition the subjects with higher than average scores for radiation anxiety (7-item) (p < 0.001), the subjects currently measuring radiation dose (p < 0.000), the subjects currently avoiding high radiation areas (p < 0.001), the subjects concerned about food radiation and production region (p < 0.001) and the subjects currently purchasing drinking water (p < 0.001) had significantly higher anxiety. The subjects responding that they were affected by harmful rumor (p < 0.001) and the subjects responding that they gained something (e.g. posttraumatic growth) through the earthquake experience (p = 0.004) also had significantly higher anxiety.

Table 5 indicates results of univariate analysis for trusted information sources and media used on respondents with current anxiety as the response variable. Based on the trusted source of information about radiation, those who answered that they trusted citizen groups or “none” had significantly more anxiety (p = 0.004, 0.001). On the other hand, those who trusted government ministries and local governments had significantly less anxiety (p = 0.002, 0.015). ‘Media use’ also yielded different anxiety levels. With regard to the type of media used to learn about radiation, those who used internet sites/blogs or word of mouth had significantly more anxiety (p = 0.012, 0.003), but those who used private local television broadcasts had significantly less anxiety (p = 0.003).

Table 6 shows the results of multiple regression analysis, with current anxiety as the objective variable, and trust in government ministries (Model 1), trust in local government (Model 2), and trust in citizen groups (Model 3) as explanatory variables. The results were different for each of the explanatory variables.

**Table 6:** Results of multiple regression analysis with current anxiety as the outcome variable and trusted information source as the explanatory variable.

	Model 1				Model 2				Model 3			
	n = 781				n = 784				n = 784			
	Trust in government ministries				Trust in local government				Trust in Citizen groups			
	β	95% Confidence Interval			β	95% Confidence Interval			β	95% Confidence Interval		
		Lower limit	Upper limit			Lower limit	Upper limit			Lower limit	Upper limit	
Trust in government ministries	-.094	-.162	-.026	**								
Trust in local government					-.071	-.140	-.003	*				
Trust in Citizen groups									.119	.051	.187	**
Age (Prime-Middle-Elderly)	-.024	-.100	.052		.007	-.064	.077		-.028	-.104	.048	
Sex	.004	-.066	.075		-.007	-.083	.070		.018	-.053	.089	
Evacuation area	.087	.014	.159	*	.087	.014	.159	*	.089	.017	.162	*
High knowledge	-.053	-.122	.017		-.049	-.118	.021		-.053	-.123	.016	
Residing at home	-.049	-.122	.024		-.044	-.117	.029		-.040	-.112	.033	
No children	-.077	-.147	-.008	*	-.076	-.146	-.007	*	-.073	-.143	-.004	*
Working	-.080	-.156	-.003	*	-.073	-.150	.004	+	-.080	-.157	-.004	*
Not healthy	.176	.106	.245	**	.182	.112	.251	**	.185	.116	.253	**
High health literacy	-.073	-.142	-.005	*	-.080	-.149	-.012	*	-.066	-.134	.003	+
R^2^	.078				.075				.084			

** P < .01, * p < .05, + p < .10

Those who trusted information released by national government ministries and local government had significantly lower anxiety than those who did not select these sources. Those who trusted information released by citizen groups had a significantly higher level of anxiety compared to those who did not select this source.

Table 7 shows the results of multiple regression analysis, with current anxiety as the objective variable, and use of private local broadcast television (Model 1), use of internet sites/blogs (Model 2), and use of word of mouth (Model 3) as explanatory variables. The results were also different for each of the explanatory variables.

**Table 7:** Results of multiple regression analysis with current anxiety as the outcome variable and media used as the explanatory variable.

	Model 1				Model 2				Model 3			
	n = 784				n = 782				n = 782			
	Used local broadcast				Used internet sites				Used word of mouth			
	β	95% Confidence Interval			β	95% Confidence Interval			β	95% Confidence Interval		
		Lower limit	Upper limit			Lower limit	Upper limit			Lower limit	Upper limit	
Private local broadcast television	-.090	-.159	-.021	*								
Internet sites/blogs					.102	0.033	.171	**				
Word of mouth									.048	-.021	.117	
Age (Prime-Middle-Elderly)	-.021	-.097	.056		.000	-0.076	.077		-.012	-.088	.064	
Sex	.000	-.071	.070		.014	-0.057	.084		.004	-.066	.075	
Evacuation area	.078	.005	.151	*	.094	0.021	.166	*	.085	.012	.158	*
High knowledge	-.053	-.123	.016		-.061	-0.131	.009	+	-.053	-.123	.017	
Residing at home	-.048	-.121	.025		-.039	-0.112	.034		-.047	-.120	.026	
No children	-.080	-.150	-.011	*	-.079	-0.148	-.009	*	-.072	-.142	-.002	*
Working	-.081	-.158	-.004	*	-.083	-0.160	-.007	*	-.080	-.157	-.003	*
Not healthy	.180	.111	.250	**	.182	0.113	.252	**	.177	.108	.247	**
High health literacy	-.079	-.148	-.010	*	-.071	-0.140	-.003	*	-.070	-.139	-.001	*
R^2^	.078				.080				.072			

** p < .01, * p < .05, + p < .10

Those who used private local broadcast television had significantly lower anxiety compared to those who did not select this source. Those who used internet sites/blogs had significantly higher anxiety compared to those who did not select this source.

## Discussion

This study examined how levels of anxiety about health due to radiation exposure were related to the trusted sources of information about radiation and type of media used for information about radiation among residents in four areas of Fukushima Prefecture.

Despite its limitations, the present study is quite novel in that, even though the disaster occurred over seven and a half years ago, no other similar surveys have been published on information and health-related anxiety among the residents of Fukushima (including those in the evacuation area). In addition, our study explored the association between media and anxiety about radiation with a particular focus on the differences in reporting on Fukushima between the national mass media and local Fukushima mass media.

We found that those who trusted private volunteer organizations such as citizen groups as information sources had significantly higher anxiety. One reason for this may be that some citizen groups highlighted a sense of the dangers of radiation in general. For example after the Fukushima nuclear plant accident, the anti-nuclear energy movement was energized, and scientists and citizen groups associated with this movement wielded influence by publicizing their own arguments and theories on the internet [30].

Unlike public organizations and media institutions, many blog writers lack the obligation and responsibility to communicate accurate information. Confirmation or verification of the information prior to sharing is rare, and thus they cannot be considered highly credible sources [31, 32]. Baseless rumors and conspiracy theories spread very quickly on the internet [33], which may also explain those who used the internet for their information had higher levels of anxiety.

Anxiety levels were lower in those who trusted the government as a source of information about radiation. The low levels of anxiety in those who trusted the government are almost consistent with the findings of a previous research [24].

As an administrative organization involved directly in residents’ daily lives, local governments are responsible for implementing radiation countermeasures on-site. In addition, each local government releases the results of air and food radiation testing on their home pages.

Those who used private local broadcast television as a source of information also had significantly lower anxiety. It may be that by conveying information based on detailed local data in a way that addressed resident concerns, private local television stations could have reduced the anxiety levels among those who used them as a source of information. In the case of the Great East Japan Earthquake, the local media, including newspapers, reportedly played an extremely important role through its original reporting that addressed local needs [34].

In this study those who used word of mouth didn’t have significantly higher levels of anxiety. But there are two studies that found the word of mouth was associated with higher risk perception or anxiety, one conducted in 2011[20] and one conducted in 2013[26]. This study, conducted in 2016 may reflect a decrease in rumors and an increase in test results and more detailed information.

Multiple regression analysis revealed that those who were in the evacuation area and those who responded that their health status was not so good had significantly higher levels of anxiety.

It is generally known that radiation doses in the evacuation area are relatively high, and that health-related anxiety would be understandably amplified when an individual’s health status is poor. Meanwhile, those with no children, those who are working, and those with higher health literacy scores had significantly lower levels of anxiety.

### Limitations

This study has some limitations. First, because of its cross-sectional design, causation could not be established. For example, it is possible that, rather than anxiety being low because participants trusted government ministries as a source of information, there were people who trusted the government as a source of information because their anxiety levels were low. Second, the respondents may have included a disproportionate number of those inclined to be relatively more cooperative with a Fukushima Medical University survey. Thus, there may have been fewer responses from people who do not trust authorities. Third, because respondents tended to be relatively older, our study population included fewer users of the internet, especially SNS, which represented a limitation to understanding the actual conditions in this area. Finally, those with poor physical or mental health are generally less likely to respond to a survey, which may have influenced the overall results.

There is a possibility that the difference in interpretation and data used will vary depending on the intended audience. Local mass media responds to the local residents’ demand and national mass media responds to the people in many parts of the country. In addition, there are many citizen groups that respond to the demand of those who are not satisfied with the information coming from sources of authority, including commonly available mass media. Furthermore, information might be shaped for commercial purposes such as highlighting sensationalism for popular sales. Headlines around the globe indicate that there are many sources of information on the internet that promulgate misinformation, fake news, and harmful rumors[35, 36] along with trusted experts' scientific information [37, 38].

### Implications for further study

In this study, it was found that the significant relationship to anxiety varies depending on the sources of trust and media used. In a nuclear disaster, the raw data is difficult for lay people to interpret. People often rely, instead, on the interpretation of others. However, the interpretations of government, local government, mass media, citizen groups and others may differ from one another. Nuclear issues are not just a matter of science, but are sometimes shaped by political factors which influence interpretation. In addition, even the choice of data and information might be different.

People may well need help and guidance identifying reliable sources of information. Media literacy education, popular in the U.S. [39, 40] may be useful in schools and help prepare the next generation to identify legitimate sources of information. Choosing reliable information from mass media and the internet is critically important for people facing nuclear disaster.

Health literacy is also very important. In this study, those in the upper group of health literacy scores tended to be less anxious. Health literacy is determined by interactions–such as those between scientists and the lay public, between health professionals and patients. Professionals can improve the health literacy of the public by ‘translating’ health information into everyday language and avoiding jargon and complex mathematical terms.

When nuclear accidents requiring high-level expertise occurred, scientific knowledge and medical knowledge of media managers, reporters and program makers inside the mass media institutions proved to be insufficient [41, 42]. At the same time, the scientists and scholars did not focus on providing information suitable to the public. During and after the Fukushima nuclear power plant accident, confusion occurred among those reporting through the mass media and contradictory information was also disseminated [43]. Therefore, the improvement of health literacy of the mass media and the news organizations is indispensable and could prove to be effective for dealing with the preparation for and aftermath of disasters. Clear communication supports informed policy makers, journalists, and communities.

## Conclusions

This study selected participants in a nearly random manner from throughout Fukushima Prefecture, including the evacuation area to assess the relationship between resident anxiety and sources of information. We found that levels of anxiety among residents following the Fukushima nuclear disaster were associated with the trusted information source and type of media used.

The reported results suggested that the content of information conveyed by sources and media differed. It seemed that there was a possibility that anxiety increased or decreased due to this difference. From this result, we thought that in order not to have excessive anxiety, it is necessary to be able to select correct information with media literacy from many information sources and media. Also, since the anxiety of the group with high health literacy was significantly lower, we thought that raising health literacy was effective in order not to have excessive anxiety.

The Fukushima nuclear power plant accident was the first nuclear disaster since the internet use has become widespread and mainstream. The internet created an overflow of mass reporting and information. A pressing task for our society is to bolster measures that will ensure the release and dissemination of accurate information in such circumstances, and to help users identify accurate information. We hope that the results of this study will be of use in future discussions of how best to convey and consume information when a major nuclear catastrophe occurs somewhere in the world.

## Supporting information

S1 Table Basic information on respondents and responses for all the questions (items except on Table 1 and Fig 1). (DOCX)

S1 Questionnaire Original question sheet in Japanese. (DOCX)

S2 Questionnaire Translation question sheet. (DOCX)
