# Supplementary material for: Lingering health-related anxiety about radiation among Fukushima residents as correlated with media information following the accident at Fukushima Daiichi Nuclear Power Plant
Source: PLoS One. 2019 May 31;14(5):e0217285. doi: 10.1371/journal.pone.0217285 (PMC6544244; doi:10.1371/journal.pone.0217285)
Supplement: S1 Table — (DOCX) [file pone.0217285.s001.docx]

S1 Table

Basic information on respondents and responses for all the questions (items except on Table 1 and Fig 1.)

| Item | Category | n | % |
| --- | --- | --- | --- |
| Area | Aizu | 262 | 30.4 |
|  | Nakadori | 206 | 23.9 |
|  | Hamadori | 201 | 23.3 |
|  | Evacuation area | 192 | 22.3 |
| Health status | Extremely good | 28 | 3.3 |
|  | Very good | 79 | 9.2 |
|  | Good | 287 | 33.5 |
|  | Fair | 385 | 44.9 |
|  | Not healthy | 78 | 9.1 |
| Exercise | Never | 397 | 46.6 |
|  | 1 to 3 times | 216 | 25.4 |
|  | 4 to 7 times | 121 | 14.2 |
|  | 8 to 15 times | 55 | 6.5 |
|  | More than 15 times | 63 | 7.4 |
| Sleep satisfaction | Satisfied | 273 | 31.9 |
|  | Slightly dissatisfied | 456 | 53.3 |
|  | Very dissatisfied | 110 | 12.9 |
|  | Very dissatisfied (I could not sleep at all) | 16 | 1.9 |
| Alcohol consumption | Yes | 247 | 29.1 |
|  | No | 538 | 63.4 |
|  | I used to drink, but I quit | 64 | 7.5 |
| Smoking | Yes | 173 | 20.3 |
|  | No | 532 | 62.4 |
|  | I used to smoke, but I quit | 147 | 17.3 |
| Regular health checks  at municipality, workplace | Yes | 474 | 55.1 |
| Complete physical | Yes | 143 | 16.6 |
| Individual dosimeter measurement  of external radiation | Yes | 86 | 10.0 |
| WBC internal radiation  measurement | Yes | 193 | 22.4 |
| Fukushima Health  Management Survey | Yes | 141 | 16.4 |
| Thyroid test field information session | Yes | 8 | 0.9 |
| Local physician lecture on radiation | Yes | 35 | 4.1 |
| Other lecture or information session | Yes | 61 | 7.1 |
| Radiation anxiety immediately  after nuclear accident | Not at all | 104 | 12.2 |
|  | Only a little | 130 | 15.2 |
|  | Somewhat | 315 | 36.8 |
|  | Very | 137 | 16.0 |
|  | Extremely | 170 | 19.9 |
| (5 items about health literacy) |  |  |  |
| 1: You can collect information from various sources such as newspapers, books, and the Internet. | I do not think so at all | 57 | 6.9 |
|  | Somewhat disagree | 73 | 8.8 |
|  | I cannot say either | 196 | 23.6 |
|  | Somewhat agree | 407 | 49.0 |
|  | Strongly agree | 98 | 11.8 |
| 2: You can pick out information you want from among a lot of information. | I do not think so at all | 66 | 8.0 |
|  | Somewhat disagree | 97 | 11.8 |
|  | I cannot say either | 351 | 42.6 |
|  | Somewhat agree | 261 | 31.6 |
|  | Strongly agree | 50 | 6.1 |
| 3: You can understand the information and tell people. | I do not think so at all | 90 | 10.9 |
|  | Somewhat disagree | 124 | 15.0 |
|  | I cannot say either | 378 | 45.8 |
|  | Somewhat agree | 200 | 24.2 |
|  | Strongly agree | 34 | 4.1 |
| 4: You can determine how reliable the information is. | I do not think so at all | 79 | 9.6 |
|  | Somewhat disagree | 148 | 17.9 |
|  | I cannot say either | 427 | 51.7 |
|  | Somewhat agree | 149 | 18.0 |
|  | Strongly agree | 23 | 2.8 |
| 5: Based on the information, you can decide plans and actions for health improvement. | I do not think so at all | 70 | 8.5 |
|  | Somewhat disagree | 112 | 13.6 |
|  | I cannot say either | 363 | 44.0 |
|  | Somewhat agree | 240 | 29.1 |
|  | Strongly agree | 41 | 5.0 |
| (5 items about radiation knowledge) |  |  |  |
| 1:Once the body receives radiation, it remains in the body | Yes | 282 | 33.3 |
|  | No | 371 | 43.9 |
|  | I don’t know | 193 | 22.8 |
| 2: According to international standards, as the exposure dose of radiation increases, the higher the probability of dying from cancer | Yes | 577 | 68.2 |
|  | No | 82 | 9.7 |
|  | I don’t know | 187 | 22.1 |
| 3: In the surveys on the health  effects of the second and third generation of atomic bomb survivors in Hiroshima and Nagasaki, genetic influences were not found. | Yes | 275 | 32.6 |
|  | No | 211 | 25.0 |
|  | I don’t know | 358 | 42.4 |
| 4: Once damaged by radiation, the DNA (the body of the gene) of cells cannot be repaired. | Yes | 394 | 46.9 |
|  | No | 132 | 15.7 |
|  | I don’t know | 315 | 37.5 |
| 5: According to government  standards for radioactive  materials, the radioactivity level  of general food items should not  exceed 100 Becquerel per kg | Yes | 344 | 41.0 |
|  | No | 118 | 14.1 |
|  | I don’t know | 378 | 45.0 |
| (7 items about radiation anxiety) |  |  |  |
| 1: I am worried I might suffer from  serious diseases due to the influence of  radiation in the future. | I do not think so at all | 136 | 16.2 |
|  | Somewhat disagree | 334 | 39.8 |
|  | Somewhat agree | 279 | 33.2 |
|  | I strongly think so | 91 | 10.8 |
| 2: Every time my condition gets worse, I become anxious about radiation exposure. | I do not think so at all | 293 | 34.9 |
|  | Somewhat disagree | 397 | 47.3 |
|  | Somewhat agree | 115 | 13.7 |
|  | I strongly think so | 34 | 4.1 |
| 3: I am worried that the influence of radiation will be inherited to the next generation, such as my children and grandchildren. | I do not think so at all | 148 | 17.6 |
|  | Somewhat disagree | 275 | 32.8 |
|  | Somewhat agree | 261 | 31.1 |
|  | I strongly think so | 155 | 18.5 |
| 4: Looking at reports on nuclear  power plant accidents, I become  very anxious. | I do not think so at all | 50 | 6.0 |
|  | Somewhat disagree | 194 | 23.1 |
|  | Somewhat agree | 358 | 42.6 |
|  | I strongly think so | 238 | 28.3 |
| 5: Because I lived in an area with supposedly high radiation doses, I am worried for myself as well as my children that we might be discriminated against (e.g., receive unfair treatment). | I do not think so at all | 139 | 16.9 |
|  | Somewhat disagree | 298 | 36.1 |
|  | Somewhat agree | 288 | 34.9 |
|  | I strongly think so | 100 | 12.1 |
| 6: I try not to talk to people as much as possible about being a local resident of the area. | I do not think so at all | 291 | 35.3 |
|  | Somewhat disagree | 331 | 40.2 |
|  | Somewhat agree | 140 | 17.0 |
|  | I strongly think so | 62 | 7.5 |
| 7: I have experienced conflicting opinions with my family about the effects of radiation on health. | I do not think so at all | 388 | 46.9 |
|  | Somewhat disagree | 266 | 32.2 |
|  | Somewhat agree | 124 | 15.0 |
|  | I strongly think so | 49 | 5.9 |
| Radiation dose measurement | No | 418 | 49.5 |
|  | I used to, but I quit | 349 | 41.3 |
|  | Yes | 78 | 9.2 |
| Avoiding high radiation areas | No | 263 | 31.5 |
|  | I used to, but I quit | 254 | 30.4 |
|  | Yes | 318 | 38.1 |
| Attentive to food radiation and  production area | No | 279 | 33.1 |
|  | I used to, but I quit | 305 | 36.1 |
|  | Yes | 260 | 30.8 |
| Purchase water | No | 435 | 51.6 |
|  | I used to, but I quit | 155 | 18.4 |
|  | Yes | 253 | 30.0 |
| Affected by harmful rumor | No | 344 | 40.5 |
|  | Somewhat | 304 | 35.8 |
|  | Yes | 201 | 23.7 |
| Gained something through  disaster experience | No | 360 | 44.6 |
|  | Yes | 448 | 55.5 |
| Current family structure | Single household (only yourself) | 106 | 12.4 |
|  | Couple-only household | 235 | 27.4 |
|  | Couple and unmarried children household | 222 | 25.9 |
|  | Household of unmarried children and you | 34 | 4.0 |
|  | Third generation family | 139 | 16.2 |
|  | Other | 122 | 14.2 |
| The highest educational qualification | Junior high school | 123 | 14.5 |
|  | High school | 441 | 51.8 |
|  | Junior college/vocational school | 182 | 21.4 |
|  | University/graduate school | 105 | 12.3 |
| Current residence | Owned house | 645 | 75.2 |
|  | Rented house or apartment | 111 | 12.9 |
|  | Temporary housing | 16 | 1.9 |
|  | Government subsidized housing | 33 | 3.9 |
|  | Public housing | 34 | 4.0 |
|  | Home of friend/relative | 3 | 0.4 |
|  | Other | 16 | 1.9 |
| Pre-earthquake residence | Owned house | 720 | 83.9 |
|  | Rented house or apartment | 111 | 12.9 |
|  | Home of friend/relative | 4 | 0.5 |
|  | Other | 23 | 2.7 |
| Relocation to avoid radiation | Yes | 225 | 27.6 |
|  | We moved for other reasons | 47 | 5.8 |
|  | No | 542 | 66.6 |
| (family situation of the relocation) | I and my family moved together | 117 | 52.5 |
|  | Only I moved | 8 | 3.6 |
|  | Only some family members moved | 26 | 11.7 |
|  | My family members and/or I evacuated immediately after the earthquake but quickly returned | 72 | 32.3 |
| Children at time of earthquake | We had a child(ren) under age 18 | 323 | 37.5 |
|  | We had a child(ren) over age 19 | 123 | 14.3 |
|  | (Female only) I was pregnant | 7 | 0.8 |
|  | We had a pregnant woman | 27 | 3.1 |
|  | None | 393 | 45.6 |
| Employment | Working (include self-employed and part-time workers) | 505 | 59.6 |
|  | I am on leave | 18 | 2.1 |
|  | Not working (student, househusband/wife, job seeker) | 325 | 38.3 |
| (4 items about social capital) |  |  |  |
| 1: People living in the area help  each other. | I do not think so at all | 57 | 6.9 |
|  | Somewhat disagree | 70 | 8.5 |
|  | I cannot say either | 250 | 30.2 |
|  | Somewhat agree | 396 | 47.9 |
|  | Strongly agree | 54 | 6.5 |
| 2: I can trust people living in the area | I do not think so at all | 41 | 5.0 |
|  | Somewhat disagree | 63 | 7.6 |
|  | I cannot say either | 309 | 37.4 |
|  | Somewhat agree | 360 | 43.6 |
|  | Strongly agree | 53 | 6.4 |
| 3: People living in the area greet  each other. | I do not think so at all | 31 | 3.7 |
|  | Somewhat disagree | 31 | 3.7 |
|  | I cannot say either | 115 | 13.8 |
|  | Somewhat agree | 481 | 57.7 |
|  | Strongly agree | 175 | 21.0 |
| 4: If problems occur in the area, people  work together to try to resolve the  problems. | I do not think so at all | 45 | 5.4 |
|  | Somewhat disagree | 48 | 5.8 |
|  | I cannot say either | 282 | 34.0 |
|  | Somewhat agree | 378 | 45.5 |
|  | Strongly agree | 77 | 9.3 |
| Participation in local groups | Neighborhood association, resident association | 533 | 61.9 |
|  | Regional groups such as youth group, women’s association, elderly association, PTA, child association (training group) | 191 | 22.2 |
|  | NPO, volunteer/citizen activity organization, co-operative association | 86 | 10.0 |
|  | Vocational organizations such as business association, peer association, industry group, labor union, etc. | 81 | 9.4 |
|  | Other | 19 | 2.2 |
|  | No | 215 | 25.0 |
