# Supplementary material for: Lingering health-related anxiety about radiation among Fukushima residents as correlated with media information following the accident at Fukushima Daiichi Nuclear Power Plant
Source: PLoS One. 2019 May 31;14(5):e0217285. doi: 10.1371/journal.pone.0217285 (PMC6544244; doi:10.1371/journal.pone.0217285)
Supplement: S1 Questionnaire — (DOCX) [file pone.0217285.s002.docx]

健康と情報についての調査

平成28年8月15日

1. 最近１か月間のあなたの身体的な健康状態は次のどれにあたりますか。最もあてはまるもの

１つに○を付けてください。

１　きわめて良い ２　とても良い ３　良い

４　まあまあ ５　不健康

２．あなたの生活習慣について

（１）汗がでるくらいの運動やスポーツを、１カ月に平均何回くらいしましたか。１つ選んで○を付けてください。

　　１　していない　　２　１～３回　　　３　４～７回　　４　８～15回　　５　15回より多い

（２）あなたは、ここ１か月間、（睡眠の長さに関わらず）睡眠の質に満足していますか。最もあてはまるもの１つ選んで○を付けてください。

　　１　満足している　　２　少し不満　 ３ かなり不満　 ４　非常に不満か、全く眠れなかった

（３）あなたは、お酒・アルコール*を毎日飲んでいますか。１つ選んで○を付けてください。（*ビ－ルなら小缶で２本、日本酒なら１合、焼酎なら0.7合、ウイスキ－・ワインなら小グラス２杯、以上）

　　１　はい　　　　　　 ２　いいえ　　　　　　 ３　以前飲んでいたが、やめた

（４）現在、ほぼ毎日、たばこを吸っていますか。１つ選んで○を付けてください。

　　１　吸っている　　　 ２　吸っていない　　　 ３　以前吸っていたが、やめた

**３．**あなたは東日本大震災以後に、以下のような健康診断や講習会・説明会を受けましたか。

あればいくつでも○をつけてください。

| １　自治体・職場が行う定期健康診査 |
| --- |
| ２　1に該当しない健康診断（人間ドックなど） |
| ３　個人線量計による外部被ばく線量（ガラスバッジ）の測定 |
| ４　内部被ばく線量（ホールボディカウンター）の測定 |
| ５　県民健康調査における健康診査 |
| ６　甲状腺検査出張説明会（保護者や教員向けに医大医師が学校に出張して説明会を実施） |
| ７　市町村主催の放射線関連の講演会・地元の医師による放射線や甲状腺をテーマとした講演会等 |
| ８　その他の講習会・説明会等 |

**４．**放射線に関する不安について

（１）東京電力福島第一原子力発電所の事故（以下、原発事故とします）が起きた直後、あなたの健康への放射線の影響について、どのくらい不安を感じましたか。最もあてはまるもの１つに○を付けてください。

１　全くない　　　２　少ししかない　　３　いくらか　　　４　たくさん　　５　非常に

（２）現在、あなたの健康への放射線の影響について、どのくらい不安を感じますか。最もあてはまるもの１つに○を付けてください。

１　全くない　　　２　少ししかない　　３　いくらか　　　４　たくさん　　５　非常に

**５．**もし必要になったら、病気や健康に関連した情報を、自分自身で探したり利用したりすることができると思いますか。それぞれの文章を読んで、最もあてはまるもの１つに○を付けてください。

|  | 全くそう思わない | どちらかといえばそう思わない | どちらとも言えない | どちらかといえばそう思う | 強くそう思う |
| --- | --- | --- | --- | --- | --- |
| １　新聞、本、インターネットなど、いろいろな情報源から情報を集められる。 | １ | ２ | ３ | ４ | ５ |
| ２　たくさんある情報の中から、自分の求める情報を選び出せる。 | １ | ２ | ３ | ４ | ５ |
| ３　情報を理解し、人に伝えることができる。 | １ | ２ | ３ | ４ | ５ |
| ４　情報がどの程度信頼できるかを判断できる。 | １ | ２ | ３ | ４ | ５ |
| ５　情報をもとに健康改善のための計画や行動を決めることができる。 | １ | ２ | ３ | ４ | ５ |

**６．**以下の文章で**「正しいと思う」**ものに**○**を、**「正しくないと思う**」ものに**×**を、どちらか**「分からない」**ものには**△**を（　）の中にご記入下さい。

| １　放射線を一度身体に受けるとその放射線はずっと体内に残る。………………………………**（　）** |
| --- |
| ２　国際的な基準では、放射線の被ばく量が多いほど、そのためにガンで死亡する確率も高くなる  という考え方が採用されている。…………………………………………………………………**（　）** |
| ３　広島、長崎の原爆被ばく者の二世、三世の健康影響に関する調査では、遺伝的影響は  認められていない。…………………………………………………………………………………**（　）** |
| ４　放射線でいったん傷ついた細胞のDNA（遺伝子の本体）は修復することができない。…**（　）** |
| ５　政府による放射性物質の基準値では一般食品は１kgあたり100ベクレルを超えないように  設定されている。……………………………………………………………………………………**（　）** |

**７．**原発事故による放射線の影響について、感じていることや、経験されたことについて伺います。それぞれの文章を読んで、最もあてはまるもの１つに○を付けてください。

|  |  | 全くそう思わない | あまりそう思わない | ややそう思う | とてもそう思う |
| --- | --- | --- | --- | --- | --- |
| １　将来、放射線の影響で深刻な病気にかかるのではないかと心配している。 | | １ | ２ | ３ | ４ |
| ２　体の具合が悪くなるたびに、放射線を浴びたせいではないかと不安になる。 | | １ | ２ | ３ | ４ |
| ３　放射線の影響が子どもや孫など次の世代に遺伝するのではないかと心配している。 | | １ | ２ | ３ | ４ |
| ４　原発事故に関する報道を見ると、とても不安になる。 | | １ | ２ | ３ | ４ |
| ５　放射線量が高いといわれる地域に住んでいたために、自分や子どもが他の人から差別される(不公平な扱いを受ける)不安がある。 | | １ | ２ | ３ | ４ |
| ６　その地域の住民であることを、なるべく人に話さないようにしている。 | | １ | ２ | ３ | ４ |
| ７　放射線が健康に与える影響について、家族と意見が対立して、もめた経験がある。 | | １ | ２ | ３ | ４ |

**８．**あなたは、原発事故の発生後から現在までに、つぎのようなことをしていますか。それぞれの文章を読んで、最もあてはまるもの１つに○を付けてください。

|  |  | していない | 以前はしていたが、今はしていない | している |
| --- | --- | --- | --- | --- |
| １　自宅や自宅周りなどの放射線量を測定する | | １ | ２ | ３ |
| ２　放射線量の高い場所に近づかないようにする | | １ | ２ | ３ |
| ３　食べ物の放射線量と産地に気をつける | | １ | ２ | ３ |
| ４　飲み水を購入する | | １ | ２ | ３ |

**９．**放射線について、その報道の元となる情報が、どこからもたらされたものならば信用できると思いますか? おもなものを**３つ**選び、〇をつけてください。

| １　　国際機関（国連　WHOなど）などが発表した情報 |
| --- |
| ２　　大学・研究所等の専門家が発表した情報 |
| ３　　政府・省庁が発表した情報 |
| ４　　地元新聞（福島民報、福島民友）が発表した情報 |
| ５　　全国新聞（讀賣新聞、朝日新聞、毎日新聞等）が発表した情報 |
| ６　　NHKが発表した情報 |
| ７　　地元民放テレビ（FTV、FCT、KFB、TUF）が発表した情報 |
| ８　　全国民放テレビ（フジ、日本テレビ、テレビ朝日、TBS等）が発表した情報 |
| ９　　地方自治体が発表した情報 |
| 10　　NGOなど民間のボランティア団体が発表した情報 |
| 11　　この中にはない |

**１０．**ふだんあなたは、放射線に関する情報をどこから得ていますか。以下のうちから、おもなものを**３つ**選び、〇をつけてください。

| １　　地元新聞（福島民報、福島民友） |
| --- |
| ２　　全国新聞（讀賣新聞、朝日新聞、毎日新聞等） |
| ３　　NHKテレビのニュース、番組 |
| ４　　地元民放テレビ（FTV、FCT、KFB、TUF）制作のニュース・番組 |
| ５　　全国民放テレビ（フジ、日本テレビ、テレビ朝日、TBS等）制作のニュース・番組 |
| ６　　ラジオ |
| ７　　インターネットのニュース（Yahooニュース等） |
| ８　　インターネットのニュース以外のサイト・ブログ |
| ９　　SNS（フェイスブック、ツイッター、LINE等） |
| 10　　雑誌・書籍 |
| 11　　自治体公報（県公報も含む） |
| 12　　クチコミ（知人、友人等） |
| 13　　その他 |

１１．原発事故に伴う風評被害は、暮らしに影響しましたか？

１　なし　　　　　　　　２　多少あり　　　　　　　　３　あり

２、３と答えた方、具体的にお書きください。

１２．東日本大震災は大きな負（マイナス）の体験であることは言うまでもありませんが、この体験から何か得たものはありますか？

１　　ない

２　　ある（具体的にお書きください）

**１３．**最後に、あなたご自身、あるいはご家庭の基本的なことがらについて伺います。立ち入った質問もありますが、正確な結果を出すために伺うことが必要です。よろしくお願いします。

１）あなたの性別・年齢を教えてください。

１　男　性　　　２　女　性　　　（　　　　　　　）歳

２）現在、あなたと同居している家族の構成は、つぎのどれにあたりますか。１つ選んで○を付けてください。

１　単身世帯（自分のみ）　　　　２　夫婦のみ　　　　　３　夫婦と未婚の子の世帯

４　あなたと未婚の子の世帯　　　５　三世代家族　　　　６　その他（ 　　　　 ）

３）あなたの最終学歴は次のどれですか。１つ選んで○を付けてください。

１　中学まで　　　　２　高校　　　　３　短大・専門学校　　　　４　大学・大学院

４）現在のお住まいは次のどれにあたりますか。１つ選んで○をつけてください。

１　自宅　　　　２　借家や賃貸アパート　　３　仮設住宅　　　４　借り上げ住宅

５　公営住宅　　６　知人・親戚の家　　　　７その他（　　　　　　　　　　　　　　　　）

５）震災前のお住まいは次のどれにあたりますか。１つ選んで○をつけてください。

１　自宅　　　　２　借家や賃貸アパート　　３　知人・親戚の家

４　その他　（　　　　　　　　　　　　　　）

６）あなたとあなたの家族は、放射線を避けるために、もとの住所地から転居されましたか。

１　転居した　２　別の理由で転居した　３　転居しなかった　　　　７）は飛ばしてください

７）６）で「転居した」と答えた方は、１つ選んで○をつけてください。

　　１　自分および家族がともに転居した　　２　自分のみ転居した

３　家族のみ転居した　　　　　　　　　４　自分または家族が震災直後に一時的に避難した

８）東日本大震災のとき、ご家族にはお子さん、または、妊婦さんがいましたか。

該当するもの全てに○をつけてください。

１　18歳以下の子供がいた　　　　　 ２　19歳以上の子供がいた

３（女性のみ）自分が妊娠していた　　４　家族が妊娠していた 　　５　いずれでもない

９）あなたは現在お仕事をしていますか。なお主夫・主婦の方でも、現在パートなどでお勤めの場合は
「働いている」とお答えください。１つ選んで○をつけてください。

| １　働いている（勤め・自営・パートを問いません）  ２　休職中である  ３　働いていない（学生、専業主夫・主婦、求職中を含みます） |  |
| --- | --- |

１０）あなたの今住んでいる地域の人々についてどう感じていますか。それぞれの文章を読んで、最もあてはまるものに○を付けてください。

|  | 全くそう思わない | どちらかと言えばそう思わない | どちらとも言えない | どちらかと言えばそう思う | 強くそう思う |
| --- | --- | --- | --- | --- | --- |
| １　今住んでいる地域の人々はお互いに助け合っている。 | **１** | **２** | **３** | **４** | **５** |
| ２　今住んでいる地域の人々は信頼できる。 | **１** | **２** | **３** | **４** | **５** |
| ３　今住んでいる地域の人々はお互いにあいさつをしている。 | **１** | **２** | **３** | **４** | **５** |
| ４　今住んでいる地域で問題が生じた場合、人々は力を合わせて解決し　ようとする。 | **１** | **２** | **３** | **４** | **５** |

１１）あなたは、つぎにあげる組織や団体に加入していますか。加入しているものにいくつでも○をつけてください。

１　町内会・自治会

２　青年団・婦人会・老人会、ＰＴＡ、子ども会(育成会)などの地域団体

３　ＮＰＯ、ボランティア・市民活動団体、生活協同組合などの任意団体

４　商店会・同業組合・業界団体、労働組合などの職業団体

５　その他（　　　　　　　　　 　　　　　　　　　　　　）

６　とくに加入していない

- 全体を通じて、何でも結構ですので、お書き頂ければ幸いです。

**こ**のたびは、調査にご協力頂き、ありがとうございました。
