# Supplementary material for: Lingering health-related anxiety about radiation among Fukushima residents as correlated with media information following the accident at Fukushima Daiichi Nuclear Power Plant
Source: PLoS One. 2019 May 31;14(5):e0217285. doi: 10.1371/journal.pone.0217285 (PMC6544244; doi:10.1371/journal.pone.0217285)
Supplement: S2 Questionnaire — (DOCX) [file pone.0217285.s003.docx]

Health and Information Survey

August 15, 2016

1. Which of the following best describes your physical condition in the last month?

Please select one and mark with “○.”

1. Extremely good 2. Very good 3. Good 4. Fair 5. Not healthy

2. Regarding your lifestyle,

(1) How many times a month do you exercise or play sports on average? Please select one and mark with “○.”

　1. Never 2. 1 to 3 times 3. 4 to 7 times 4. 8 to 15 times 5. More than 15 times

（2）Are you satisfied with the quality of your sleep (regardless of length) in the past month? Please select the most appropriate response and mark with “○.”

　1. Satisfied 2. Slightly dissatisfied 3. Very dissatisfied 4. Very dissatisfied (I could not sleep at all)

（3）Do you drink alcohol every day? Please select one and mark with “○.”（*Two small cans for beer, one *go* (180 mL) for sake, 0.7 *go* for shochu, and two small cups or more for whiskey/wine）

　　1. Yes　　　　　2. No　　 3. I used to drink, but I quit

（4）Do you currently smoke cigarettes almost every day? Please select one and mark with “○.”

　　1. Yes　　　 2. No　　　 3. I used to smoke, but I quit

**3．** Have you participated in the following health check-ups, seminars, or explanatory meetings since the Great East Japan Earthquake? Please select and mark with “○” (multiple answers allowed).

| 1.　Regular municipal/workplace health check-ups |
| --- |
| 2.　Other health check-ups (e.g., complete physical examination) |
| 3.　Individual dose assessment of external radiation using a dosimeter |
| 4.　WBC internal radiation measurement |
| 5.　Fukushima Health Management Survey |
| 6.　Thyroid test field information session |
| 7.　Local physician lecture on radiation |
| 8.　Other lectures or information sessions |

**4.** Regarding radiation anxiety,

（1）Just after the Tokyo Electric Power Fukushima Daiichi Nuclear Power Plant accident (hereinafter, nuclear accident), how anxious did you feel about the impact of radiation on your health? Please select the most appropriate response and mark with “○.”

1. Not at all 2. Only a little 3. Somewhat 4. Very 5. Extremely

（2）How uneasy do you feel about the impact of radiation on your health now? Please select the most appropriate response and mark with “○.”

1. Not at all 2. Only a little 3. Somewhat 4. Very 5. Extremely

**5．** Do you think you can find and use information on diseases and health on your own if you need it? Please read each statement and mark the most appropriate response with “○.”

|  | I do not think so at all | Somewhat disagree | I can not say either | Somewhat agree | Strongly agree |
| --- | --- | --- | --- | --- | --- |
| 1. You can collect information from various sources such as newspapers, books, and the Internet. | 1 | 2 | 3 | 4 | 5 |
| 2. You can pick out information you want from among a lot of information. | 1 | 2 | 3 | 4 | 5 |
| 3. You can understand the information and tell people. | 1 | 2 | 3 | 4 | 5 |
| 4. You can determine how reliable the information is. | 1 | 2 | 3 | 4 | 5 |
| 5. Based on the information, you can decide plans and actions for health improvement. | 1 | 2 | 3 | 4 | 5 |

**6．** For each of the following sentences, please put a ○ in the ( ) if you think “it is correct,” × if you think “it is not correct,” or △ if you are unsure.

| 1. Once the body receives radiation, it remains in the body.……………………（　） |
| --- |
| 2. According to international standards, as the exposure dose of radiation increases, the higher the probability of dying from cancer..……………（　） |
| 3. In the surveys on the health effects of the second and third generation of atomic bomb survivors in  Hiroshima and Nagasaki, genetic influences were not found.……………………………………………（　） |
| 4. Once damaged by radiation, the DNA (the body of the gene) of cells cannot be repaired.……………（　） |
| 5. According to government standards for radioactive materials, the radioactivity level of general food items  should not exceed 100 Becquerel per kg.………………………………………………（　） |

7. Now, we ask you about the influence of radiation caused by the nuclear accident, what you experienced, and how you feel about it. Please read each statement below and mark the most appropriate response with “○.”

|  |  | I do not think so at all | Somewhat disagree | Somewhat agree | I strongly think so |
| --- | --- | --- | --- | --- | --- |
| 1. I am worried I might suffer from serious diseases due  to the influence of radiation in the future. | | 1 | 2 | 3 | 4 |
| 2. Every time my condition gets worse, I become anxious about radiation exposure. | | 1 | 2 | 3 | 4 |
| 3. I am worried that the influence of radiation will be inherited to the next generation, such as my children and grandchildren. | | 1 | 2 | 3 | 4 |
| 4. Looking at reports on nuclear power plant accidents, I  become very anxious. | | 1 | 2 | 3 | 4 |
| 5. Because I lived in an area with supposedly high radiation doses, I am worried for myself as well as my children that we might be discriminated against (e.g., receive unfair treatment). | | 1 | 2 | 3 | 4 |
| 6. I try not to talk to people as much as possible about being a local resident of the area. | | 1 | 2 | 3 | 4 |
| 7. I have experienced conflicting opinions with my family about the effects of radiation on health. | | 1 | 2 | 3 | 4 |

8． Have you adopted the following behaviors since the occurrence of nuclear accident? For each item, please mark the most appropriate response with “○.”

|  |  | No | I used to, but I quit | Yes |
| --- | --- | --- | --- | --- |
| 1．Measure radiation doses | | 1 | 2 | 3 |
| 2．Avoid high radiation areas | | 1 | 2 | 3 |
| 3．Be attentive to food radiation and production area | | 1 | 2 | 3 |
| 4．Purchase water | | 1 | 2 | 3 |

9．Do you feel you can trust information on radiation based on the source of information (e.g., organization, group, or people)? Please choose 3 sources from the below list that you would trust and mark with “○.”

| 1. International organizations |
| --- |
| 2. Experts from universities, academic institutions, and others |
| 3. Government ministries |
| 4. Local newspapers (Fukushima Minpo and Fukushima Minyu) |
| 5. National newspapers (Yomiuri, Asahi, Mainichi, and others) |
| 6. NHK |
| 7. Private local broadcast television (FTV, FCT, KFB, TUF) |
| 8. Private national broadcast television |
| 9. Local government |
| 10 Private volunteer groups such as NGOs |
| 11. None of the above |

10. What are your sources of information on radiation? Please choose 3 out of the following responses and mark with “○.”

| 1. Local newspapers |
| --- |
| 2. National newspapers |
| 3. NHK television |
| 4. Private local broadcast television |
| 5. Private national broadcast television |
| 6. Radio |
| 7. Internet news (Yahoo, etc.) |
| 8. Internet sites and blogs other than news |
| 9. SNS (Facebook, Twitter, LINE, etc.) |
| 10. Books and magazines |
| 11. Local government publications |
| 12. Word of mouth (friends and acquaintance) |
| 13. None of the above |

11. Has the damage caused by the harmful rumor affected your life?

1. No　　　　　　　　2. Somewhat 　　　　　　　3. Yes

If you answered 2 or 3, please provide specific details.

12. While it goes without saying that the Great East Japan Earthquake disaster is characterized by significant negative experiences, have you gained something from those experiences?

1. No

2. Yes（Please provide specific details.）

13. Finally, we ask you about yourself and basic matters concerning your home and family. There are questions related to your private life, but they are necessary in order to obtain accurate results. We thank you in advance for your contribution.

1）Please tell us your sex and age.

1. Male　　　2. Female　　　 （　　　　　　　）years

2) Which of the following best describes your current family status? Please select one and mark with “○.”

1. Single household (only yourself) 2. Couple-only household 3. Couple and unmarried children household

4. Household of unmarried children and you 5. Third generation family 6. Other

3）What is the highest educational qualification that you have completed? Please select one and mark with “○.”

1. Junior high school 2. High school 3. Junior college/vocational school 4. University/graduate school

4）Which of the following is your current residence? Please select one and mark with “○.”

1. Owned house 2. Rented house or apartment 3. Temporary housing 4. Government subsidized housing

5. Public housing 6. Home of friend/relative 7. Other

5）Which of the following was your residence before the disaster? Please select one and mark with “○.”

1. Owned house 2. Rented house or apartment 3. Home of friend/relative 4. Other ( )

6）Have you and your family moved from your original address to avoid radiation?

1. Yes 2. We moved for other reasons 3. No

If you selected 3, please skip 7).

7) If you selected 1 or 2 in 6), please choose one of the following responses and mark with “○.”

　1. I and my family moved together 　2. Only I moved 3. Only some family members moved

4. My family members and/or I evacuated immediately after the earthquake but quickly returned

8）At the time of the Great East Japan Earthquake, did your family have a child/ren or pregnant woman?

Please circle all that apply.

　1. We had a child(ren) under age 18 2. We had a child(ren) over age 19

3. (Female only) I was pregnant 4. We had a pregnant woman 5. None

9) Are you currently working? Please select one and mark with “○.” (Even if you are a househusband or housewife, if you are currently working part-time, etc., please select “Working.”)

1. Working (include self-employed and part-time workers)

2. I am on leave

3. Not working (student, househusband/wife, job seeker)

10）How do you think about people in your area you live in? Please read each statement and mark the most appropriate response with “○”.

|  | I do not think so at all | Somewhat disagree | I can not say either | Somewhat agree | Strongly agree |
| --- | --- | --- | --- | --- | --- |
| 1. People living in the area help  each other. | 1 | 2 | 3 | 4 | 5 |
| 2. I can trust people living in the area. | 1 | 2 | 3 | 4 | 5 |
| 3. People living in the area greet  each other. | 1 | 2 | 3 | 4 | 5 |
| 4. If problems occur in the area, people  work together to try to resolve the  problems. | 1 | 2 | 3 | 4 | 5 |

11）Are you enrolled in the following organizations or groups?

Please mark as many responses as appropriate with “○.”

1. Neighborhood association · resident association

2. Regional groups such as youth group, women’s association, elderly association, PTA, child association (training group)

3. NPO, volunteer/citizen activity organization, co-operative association

4. Vocational organizations such as business association, peer association, industry group, labor union, etc.

5. Other（　　　　　　　　　 　　　　　　　　　　　　）

6. No

◇ Please provide any additional comments below.

　Thank you for your cooperation.
